# Robust CAR-T memory formation and function via hematopoietic stem cell delivery

**DOI:** 10.1371/journal.ppat.1009404

**Published:** 2021-04-01

**Authors:** Anjie Zhen, Mayra A. Carrillo, Wenli Mu, Valerie Rezek, Heather Martin, Philip Hamid, Irvin S. Y. Chen, Otto O. Yang, Jerome A. Zack, Scott G. Kitchen

**Affiliations:** 1 Division of Hematology/Oncology, Department of Medicine, David Geffen School of Medicine at UCLA, Los Angeles, California, United States of America; 2 UCLA AIDS Institute and the Eli and Edythe Broad Center of Regenerative Medicine and Stem Cell Research, David Geffen School of Medicine at UCLA, Los Angeles, California, United States of America; 3 Department of Microbiology, Immunology, and Molecular Genetics, University of California, Los Angeles, Los Angeles, California, United States of America; 4 Department of Infectious Disease, David Geffen School of Medicine at UCLA, Los Angeles, California, United States of America; Vaccine Research Center, UNITED STATES

## Abstract

Due to the durability and persistence of reservoirs of HIV-1-infected cells, combination antiretroviral therapy (ART) is insufficient in eradicating infection. Achieving HIV-1 cure or sustained remission without ART treatment will require the enhanced and persistent effective antiviral immune responses. Chimeric Antigen Receptor (CAR) T-cells have emerged as a powerful immunotherapy and show promise in treating HIV-1 infection. Persistence, trafficking, and maintenance of function remain to be a challenge in many of these approaches, which are based on peripheral T cell modification. To overcome many of these issues, we have previously demonstrated successful long-term engraftment and production of anti-HIV CAR T cells in modified hematopoietic stem cells (HSCs) in vivo. Here we report the development and *in vivo* testing of second generation CD4-based CARs (CD4CAR) against HIV-1 infection using a HSCs-based approach. We found that a modified, truncated CD4-based CAR (D1D2CAR) allows better CAR-T cell differentiation from gene modified HSCs, and maintains similar CTL activity as compared to the full length CD4-based CAR. In addition, D1D2CAR does not mediate HIV infection or stimulation mediated by IL-16, suggesting lower risk of off-target effects. Interestingly, stimulatory domains of 4-1BB but not CD28 allowed successful hematopoietic differentiation and improved anti-viral function of CAR T cells from CAR modified HSCs. Addition of 4-1BB to CD4 based CARs led to faster suppression of viremia during early untreated HIV-1 infection. D1D2CAR 4-1BB mice had faster viral suppression in combination with ART and better persistence of CAR T cells during ART. In summary, our data indicate that the D1D2CAR-41BB is a superior CAR, showing better HSC differentiation, viral suppression and persistence, and less deleterious functions compared to the original CD4CAR, and should continue to be pursued as a candidate for clinical study.

## Author summary

Engineering T cells with anti-HIV chimeric antigen receptors (CAR) has emerged as a promising strategy to control HIV infection through a genetic vaccination strategy. Here we report a novel CAR-based approach targeting HIV infection using the genetic modification of blood forming hematopoietic stem cells (HSCs). This novel CAR approach uses a modified HIV receptor molecule (the primary HIV receptor CD4) as well as anti-HIV agents to modify HSCs to allow them to develop into cells that are protected from HIV infection and target HIV infected cells for the life of the individual. We found this latest generation of CARs successfully modified and allowed in vivo engraftment that resulted in the development of effective anti-HIV CAR T cells with robust memory formation and viral control. Our study highlights the identification of a next-generation CAR molecule that protected cells from infection, targeted and reduced HIV burdens, and serves as an ideal developmental candidate for further clinical studies.

## Introduction

Virus-specific T cell adaptive immunity is key to the elimination of HIV-1-infected cells and is crucial for any strategic approach to achieve cure of infection. Latently infected cells persist even after decades of cART-dependent suppression of plasma viremia, thus precluding viral eradication by cART treatment alone [1]. Attempts to purge HIV-1 reservoirs also have had limited to little success in clinical trials [2, 3]. To date, there have been three documented therapeutic cures, after allogeneic bone marrow transplantation from CCR5Δ32 homozygous donors [4–6]. However, allogeneic bone marrow transplantation is unlikely to be broadly translatable due to high mortality risk of this procedure, failures to repeat prior successes, and difficulties in finding matching donor bone marrow with homozygous CCR5Δ32 mutation [7, 8]. These studies highlight the fact that although CCR5 elimination or other genetic strategies are essential for HIV-1 eradication, allogeneic HSC transplantation is not broadly applicable, underscoring the need to enhance host anti-viral responses in order to persistently suppress viral replication arising from residual, latently-infected cells.

The use of chimeric antigen receptor (CARs) to redirect T cell immunity against HIV-1 represents a highly promising gene therapy approach that can be used in HIV-1 infected individuals irrespective of Human Leukocyte Antigen (HLA) type. CARs recognize target cells through direct binding to specific cell surface antigens and thus are HLA-unrestricted and redirect both CD4+ and CD8+ T cells, bypassing a major limitation for T cell immunotherapies [9]. While recently widely applied for cancer, the first CAR clinical trials were for HIV-1 infection, testing a CAR composed of the CD4 extracellular domain linked to the intracellular CD3-ζ chain signaling domain (CD4CAR), utilizing CD4 binding to HIV-1 Envelope (Env) for targeting and killing of HIV-1 infected cells by redirected peripheral T cells [9]. Initial clinical trials with CD4CAR modified T cells showed excellent safety but revealed limited antiviral efficacy *in vivo* [10, 11]. Follow-up over a decade indicated only low-level persistence of transduced cells but continued clinical safety [12]. The limited efficacy was likely due to factors such as: suboptimal T-cell processing/handling, low levels of HIV-1 antigen during ART treatment, CAR T cell dependence on antigenic stimulation for proliferation/maturation, and potential enhancement of cellular HIV-1 infection by the CD4 portion of the CAR [13–15]. We and others have shown that protection of CD4CAR modified cells from viral entry facilitated by the CAR is essential in order to ensure T-cell functionality and survival [13, 14, 16].

The success of peripheral T cell CAR gene therapy to target HIV-1 and various malignancies has been limited by inadequate function, persistence, and trafficking of transduced T cells [17]. Hematopoietic stem cell (HSC) gene therapy has the potential to overcome many of these issues and offers the lifelong generation of physiologically functional CAR T cells *in vivo*. Autologous HSCs are capable of long-term engraftment with substantially lower morbidity and mortality than allogeneic transplantation. HSC based gene therapy allows for transduced cells to undergo normal immune developmental mechanisms, including thymic selection, which eliminates potentially self-reactive T cells and increases the potential for the development of immunological memory [16, 18, 19]. We have previously demonstrated that autologous HSCs modified with a gene for a T cell receptor (TCR) against HIV-1 or melanoma antigen [19, 20], or an anti-HIV-1 CAR [18], resulted in successful differentiation of modified T cells that resulted in significant suppression of HIV-1 replication/cancer growth in humanized bone marrow-thymus-liver (BLT) mice. Most recently, we demonstrated the feasibility, safety, and *in vivo* SHIV suppression of the HSC-based CAR approach in non-human primates (NHPs) [16]. Importantly, we found CAR-HSCs transplanted animals have lower viral rebound after release of cART as compared to control animals and CAR expressing cells were found in multiple lymphoid tissues, resulting in decreased tissue viral RNA levels and protection of CD4+ T cells in the gut, which is one of the primary replication and reservoir sites for HIV-1.

Addition of costimulatory domains in tandem with CD3ζ, such as the signaling domains of CD28 or CD137 (4–1BB), has been shown to enhance *in vivo* CAR T cell function and persistence [21–23]. These second-generation CAR T cells have been confirmed to mediate potent anti-leukemia responses of peripheral T cells in clinical trials [24, 25]. While CAR T cells containing the CD28 costimulatory domain generally undergo more intense proliferation immediately following stimulation, peripheral CAR T cells with the 4-1BB costimulatory domain show longer persistence and more sustained anti-tumor effect [21, 26, 27]. However, it is completely unknown if addition of costimulatory domains would improve HSC-based CAR therapy. In this report, we examine ways to effectively enhance HSC-based HIV-targeting CAR cell function. We developed an optimized CD4-based CAR that is resistant to non-specific receptor activation as well as does not mediate viral entry. We examined the effects of the costimulatory domains CD28 and 41BB on the engraftment and anti-HIV-1 efficacy of HSCs based CAR therapy *in vivo*. We found that the resultant CARs facilitated greater engraftment, T cell production, antiviral function compared to the original CD4CAR, as well as uniquely generated memory T cell responses *in vivo*, suggesting potentially greater clinical efficacy with this improved stem cell-based approach.

## Results

### Truncated form of CD4CAR does not mediate HIV-1 infection

**Fig 1:**
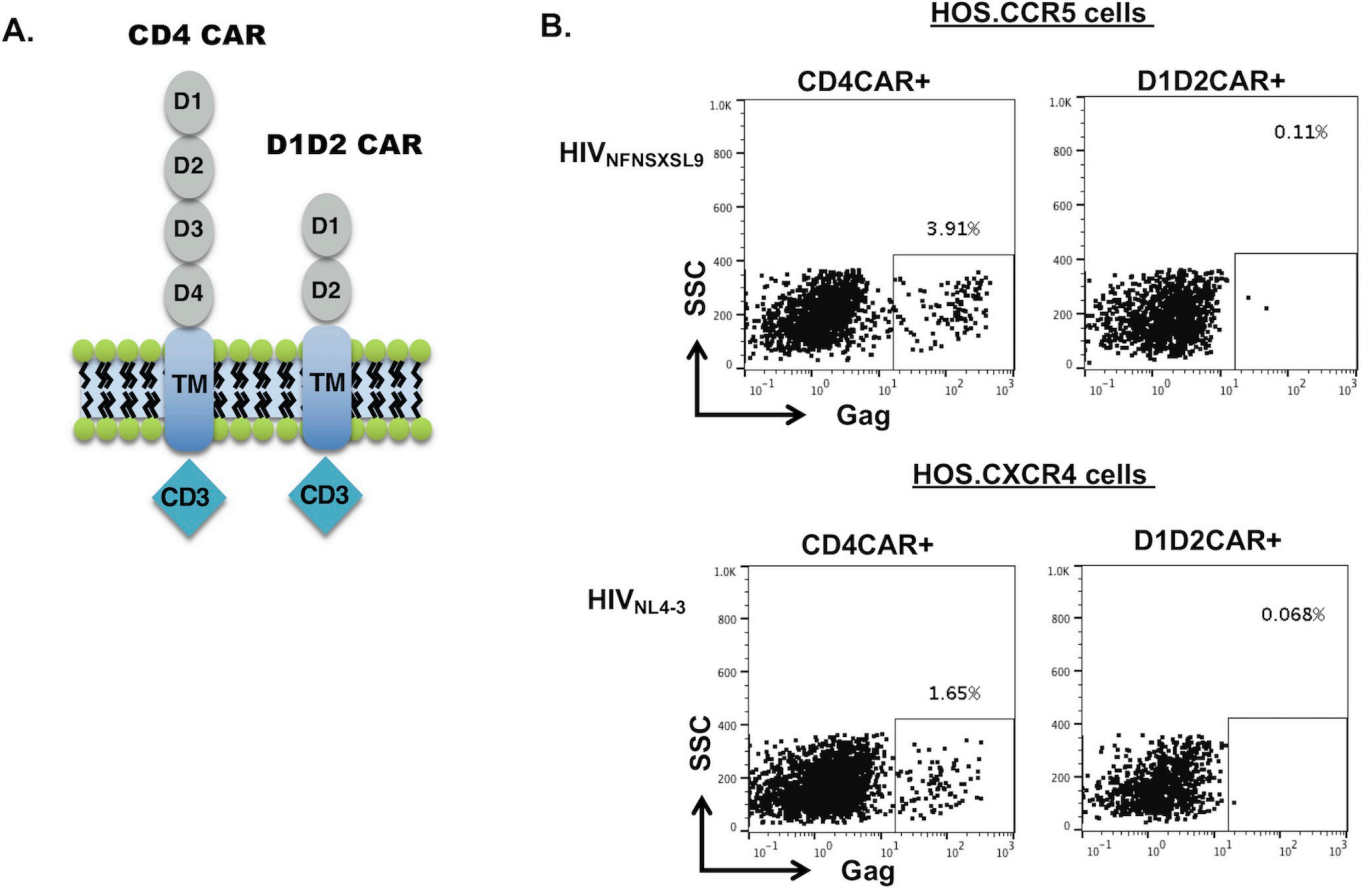
D1D2CAR does not mediate HIV infection. A. Schematic review of full length CD4CAR and truncated D1D2CAR molecule. B. D1D2CAR does not mediate HIV infection. CD4 negative HOS cells that express either CCR5 (HOS.CCR5) or CXCR4 (HOS.CXCR4) were transduced with either CD4CAR or D1D2CAR and infected with either R5 tropic HIV_NFNSXSL9_ or X4 tropic HIV_NL4-3_ for 3 days. Infection of CAR+ cells (GFP+) were analyzed by intracellular staining of Gag and flow cytometry.

One undesirable feature of the CD4CAR used in our previous studies and clinical trials is that the receptor itself can mediate HIV-1 infection [14, 15, 28]. The CD4 molecule is comprised of four extracellular domains, denoted D1-D4 [29]. The D1 domain, the most distal from the transmembrane domain, comprises the HIV-1 envelope binding region (**Fig 1A)** [30, 31]. HLA class II binding sites are found in primarily in D1, D2 domain [32], and the D4 domain mediates interleukin-16 (IL-16) binding and CD4-CD4 dimerization [33]. IL-16 is an immune-modulatory cytokine that primarily functions as a chemoattractant for CD4-bearing cells at sites of inflammation [34]. In the context of a CD4-based CAR, IL-16 binding in the D4 domain can lead to possible non-specific signaling of CD4CAR via IL-16 [33]. The D3 domain of CD4 receptor also plays an important role in TCR:CD4 complex formation and TCR stimulation [35]. To address these issues and create a CAR that does not cross-react with IL-16, has decreased interaction with HLA class II and the endogenous TCR, and potentially increases the safety of the molecule, we created a truncated CD4 CAR molecule by deleting the D3 and D4 domains, leaving only the D1D2 domains of CD4 that will allow HIV-1 envelope recognition (D1D2CAR) (**Fig 1A**).

To elucidate CD4CAR mediated HIV-1 infection, we transduced HOS.CCR5 or HOS.CXCR4 cells that are not susceptible to HIV-1 due to the lack of CD4 expression with lentiviral vectors expressing the CD4CAR or D1D2CAR and then infected them with either R5 tropic HIV_NFNSXSL9_ or X4 tropic HIV_NL4-3_. Infection of CAR+GFP+ cells were analyzed by intracellular staining of Gag followed by flow cytometry. As shown in **Fig 1B**, expression of CD4CAR resulted in successful infection of HOS CCR5 or HOS CXCR4 cells by R-5 tropic HIV_NFNSXSL9_ or X-4 tropic HIV_NL4-3_. Expression of the D1D2CAR did not render cells permissive to HIV-1 infection **(Fig 1B)**, indicating that this truncated CAR does not allow viral entry despite its ability to bind to HIV-1 Envelope. These results suggest that the D1D2CAR has an added safety aspect in its use, not facilitating HIV-1 infection like the full length CD4CAR does when expressed on the surface of a cell.

### The D1D2CAR functions similarly to full length CD4CAR in T cells

**Fig 2:**
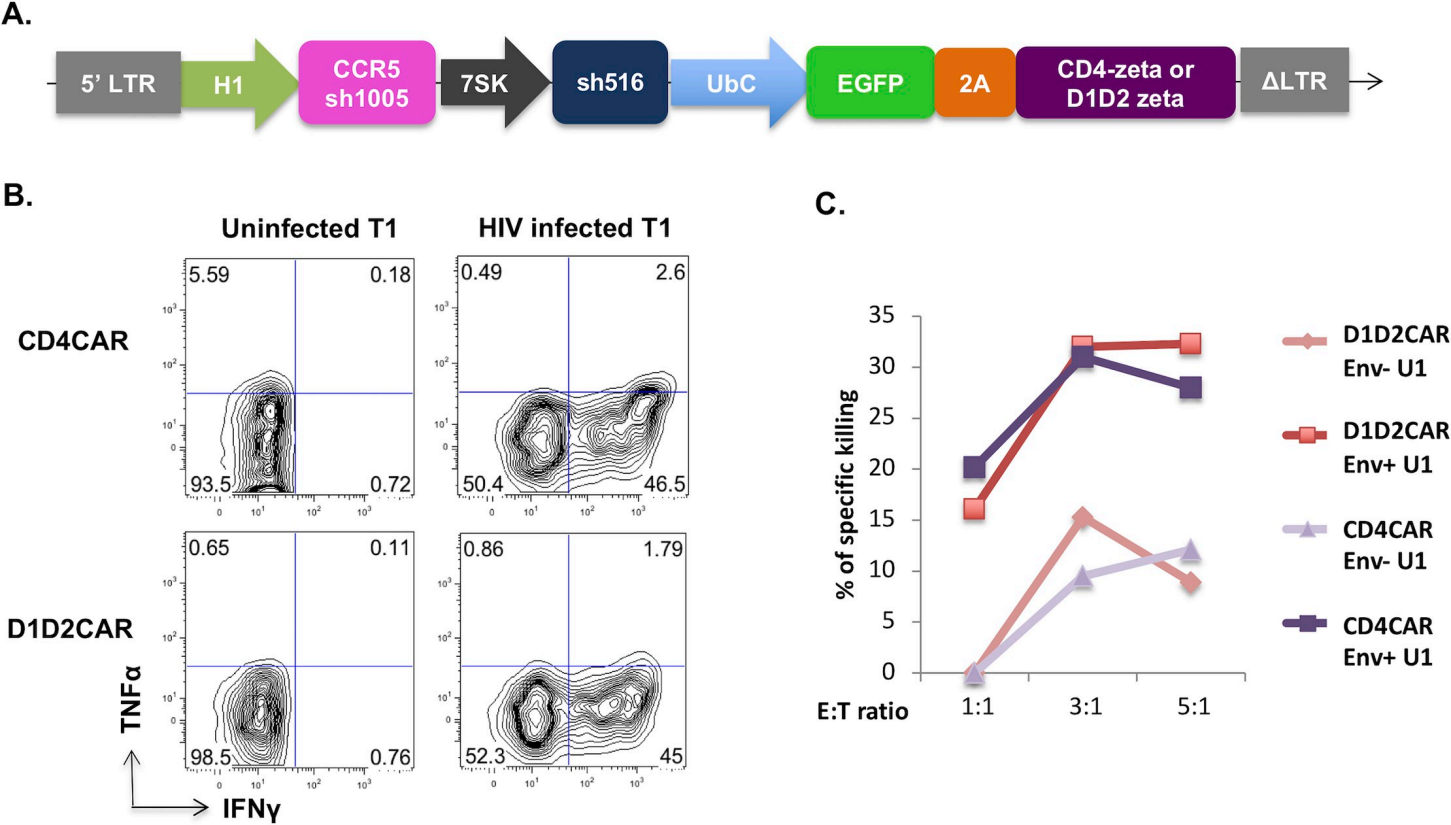
D1D2CAR CAR T cells has similar *in vitro* function compared to CD4CAR T cells. A. Schematic review of protective CD4CAR and D1D2CAR vector. B. Cytokine production of CD8 CAR T cells co-incubated with uninfected or HIV infected T1 cells. Sorted primary CD8 T cells were stimulated with anti-CD3/CD28 for 2 days and transduced with either protective CD4CAR or D1D2CAR lentiviral vector. Two days after transduction, CAR T cells were co-incubated at with either uninfected or HIV-1 infected T1 cells at 1:10 ratio overnight. Afterwards, cells were treated with GolgiPlug for 6hours before intracellular staining of cytokines and flow cytometry analysis. C. Specific killing of unstimulated (Env-) and stimulated U1 cells (Env+) by CD4CAR or D1D2CAR CD8 T cells *in vitro*. Primary CD8 T cells were transduced with protective CD4CAR or D1D2CAR lentiviruses. 2 days after transduction, CAR T cells were co-incubated with pre-stained PMA stimulated (Env+) or unstimulated (Env-) U1 cells at 1:1, 3:1, 5:1 effector to target ratio for 16 hours. Afterwards, cells were stained with zombie violet fixable viability kit and analyzed by flow cytometry. % specific killing was calculated by (%live gag+ U1 cells without CAR cell—%live gag+ U1 cells with CAR cells)/ %live gag+ U1 cells without CAR cells.

Previously, to prevent CD4CAR mediated HIV-1 infection, we have included anti-HIV-1 reagents in our lentiviral vectors to protect CD4CAR expressing cells from HIV-1 infection [13, 16]. One lentiviral vector contained the CD4CAR molecule as well as two antiviral genes: a short hairpin RNA (shRNA) targeting CCR5 coreceptor expression and a shRNA that targets HIV-1 RNA for degradation [13]. We have shown previously that the presence of these antiviral shRNAs protected CAR T cells from infection [13]. To compare the function of D1D2CAR T cells with CD4CAR T cells, we constructed lentiviral vectors that contain both the anti-HIV-1 reagents as well as the D1D2CAR (which itself does not permit infection) **(Fig 2A)**. This will allow protection of all CAR-modified progeny cell lineages expressing endogenous CD4, including mature CD4+ T lineage cells. Although D1D2CAR does not mediate HIV infection on cells that do not express endogenous CD4, it does not have protective effects for CD4+ progenitor and T cells against HIV infection; therefore, anti-HIV protection with shRNAs is still necessary. In this study, for fair comparison, all CAR lentiviruses used in *in vitro* or *in vivo* studies contain anti-CCR5 and anti-HIV shRNAs. To assess if the D1D2CAR transduced T cells respond to HIV-1 infection, we co-incubated D1D2CAR transduced CD8+ T cells with HIV-infected or uninfected T1 cells and measured production of intracellular cytokines interferon-γ (IFN-γ) and tumor necrosis factor alpha (TNF-α). As shown in **Fig 2B**, both CARs exhibit similar expression levels of IFN-γ and TNF-α production in response to HIV-1 infected target cells. We further examined specific cytotoxic activity of CD4CAR-expressing T cells versus D1D2CAR-expressing T cells by co-incubating vector transduced CD8+ T cells with either target HIV-1 envelope expressing cells (Env+ U1 cells) or control cells (Env- U1 cells). As shown in **Fig 2C**, both CD4CAR and D1D2CAR show comparable cytotoxic activity at multiple effector to target ratios. These results indicate that the D1D2CAR is functionally capable of inducing antiviral responses at a level similar to the full length CD4CAR.

### D1D2CAR does not affect T cell differentiation and TCR expression

We have previously demonstrated that CAR modified HSCs can successfully differentiate into functional T cells that recognize HIV-1 infected cells in both a humanized mouse model [13] and in NHPs [16]. Interestingly, we also observed that expression of full length CD4CAR led to shut down of endogenous TCR rearrangement and surface expression in roughly half of the differentiated CAR-expressing T cells [13]. This appears to be caused by the extracellular CD4 domain’s interaction of the CD4CAR with HLA class II expressed by the thymic stroma, which permits positive selection and shut down of endogenous TCR rearrangement.

**Fig 3:**
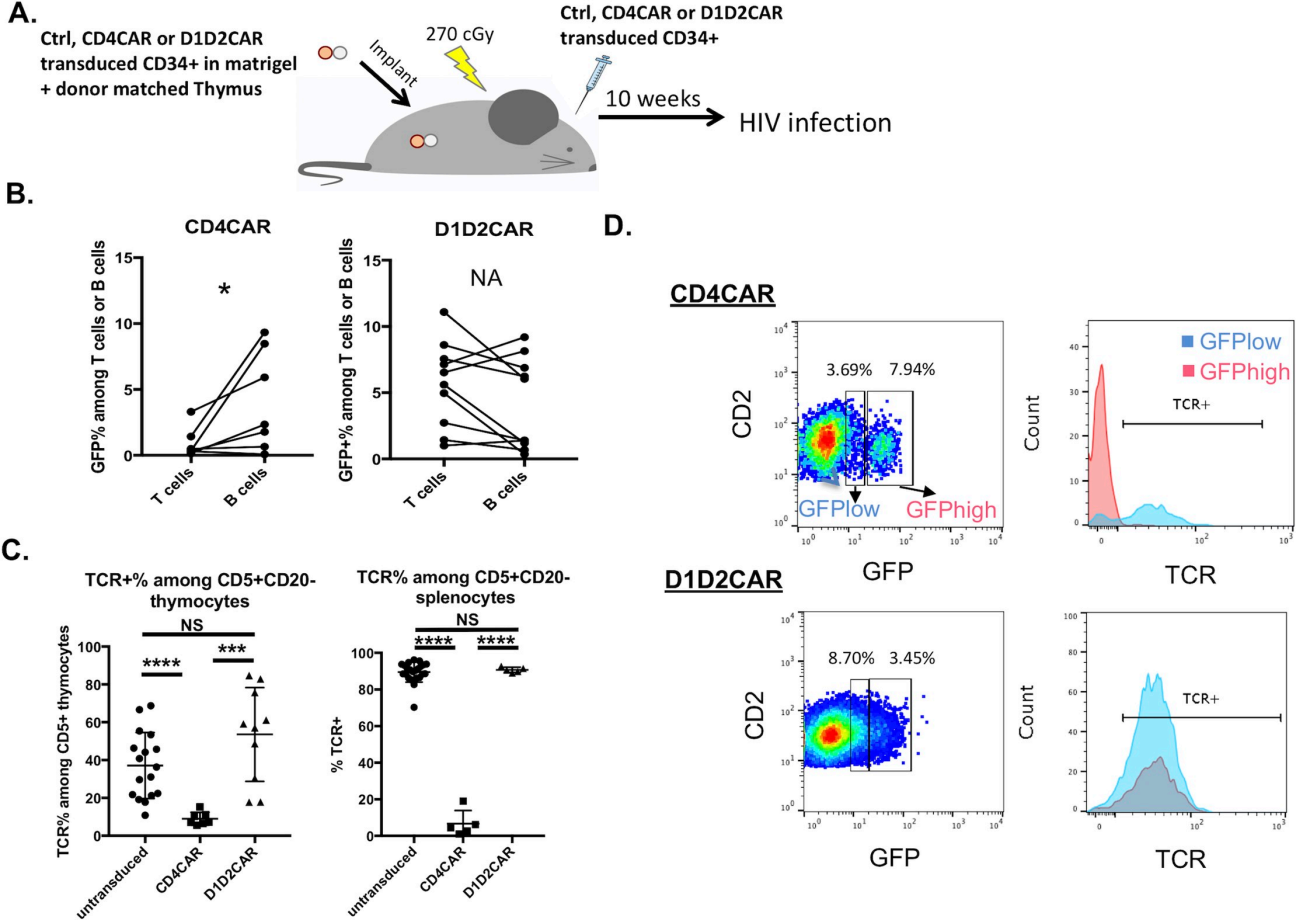
D1D2CAR does not affect T cell differentiation and TCR gene arrangement and expression. A) Humanized BLT mice were constructed with donor matched thymus and liver derived CD34+ cells that were either unmodified or transduced with either protective CD4CAR or D1D2CAR lentiviruses. 10 weeks after transplant and immune reconstitution, mice were infected with 200ng R5 tropic HIV_NFNSXSL9_. B) GFP(CAR) expression among peripheral human “classic” CD3 T cells (CD45+CD3+) and B cells (CD45+CD19+) prior to HIV infection. * p<0.01 by Wilcoxon matched-pairs signed rank test. C) TCR+% among CAR- or CD4CAR+, D1D2CAR+ thymocytes or splenocytes. * p<0.01, **p<0.001, ***p<0.0001 by Mann-Whitney test. D) TCR expression in GFPhigh and GFPlow expressing CAR T cells.

To examine if D1D2CAR also suppresses endogenous TCR expression, we constructed humanized bone marrow-liver-thymus (BLT) mice with HSCs transduced with lentiviral vectors expressing either the CD4CAR or D1D2CAR as described above. Mice were infected with HIV-1 10 weeks after surgery, following confirmation of humanization **(Fig 3A)**. Percentages of CAR+GFP+ cells among “classic” CD45+CD3+ T cells and CD45+CD19+ B cells from human peripheral blood mononuclear cells (PBMCs) were assessed before HIV-1 infection. Humanized mice receiving the full length CD4CAR transduced HSCs developed a higher percentage of CAR+ B cells as compared to CAR+ T cells in the peripheral blood, suggesting a preferential differentiation of CAR B cells or impaired CAR T cell differentiation **(Fig 3B)**. In contrast, humanized mice with D1D2CAR transduced HSCs showed similar levels of CAR expressing T cells and B cells within the same mouse, thus did not show a skewing of hematopoiesis towards B cells. Interestingly, when we examined TCR expression among thymocytes and T cells in the spleen (CD45+CD2+CD5+CD20- cells), CD4CAR transduced mice had significantly reduced expression of TCR among CAR+ thymocytes and spleen T cells **(Fig 3C)**, similar to what we have reported previously [13]. In contrast, D1D2CAR expression did not affect endogenous TCR expression on CAR T cells. In addition, similar to our previous report, high level expression of the CD4CAR on T cells led to shut down of TCR and CD3 expression **(Fig 3D)**. In contrast, expression of D1D2CAR did not affect TCR and CD3 expression on T cells regardless of the expression level. These results strongly suggest that the truncated D1D2CAR does not affect endogenous TCR rearrangement during thymopoiesis, preventing hematopoietic skewing towards the B cell lineage and leading to normal CAR T cell development. This may due to the deletion of the D3 and D4 regions that are required for signaling via HLA-II binding, which in turn affects positive and/or negative selection in the thymus [13, 35].

### HSCs based D1D2CAR therapy suppresses HIV-1 replication *in vivo*

**Fig 4:**
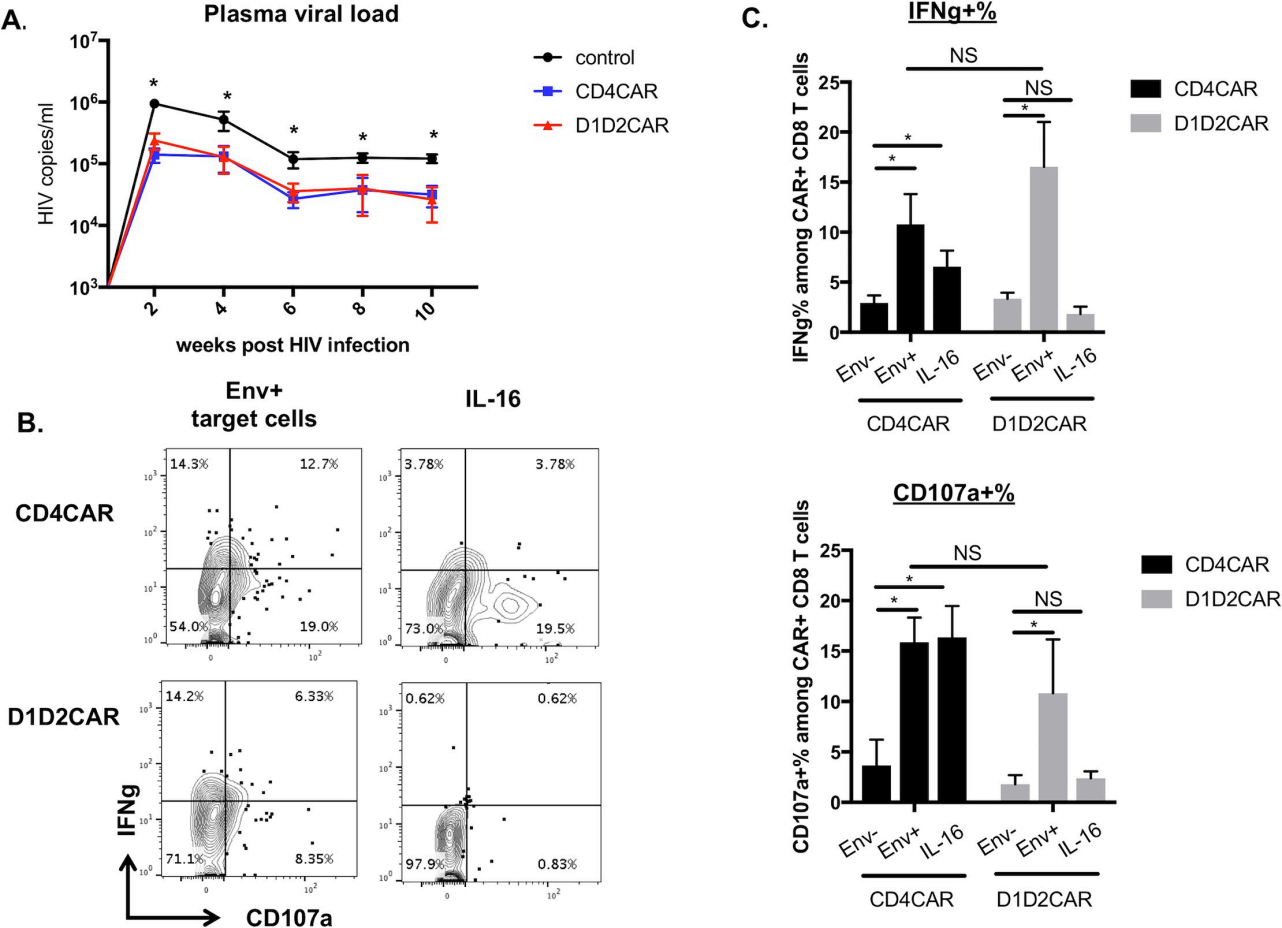
Stem cell based D1D2CAR therapy suppressed HIV replication *in vivo*. A) Plasma HIV viral load of mice that were transplanted with protective CD4CAR or D1D2CAR transduced CD34+ cells or control CD34+ cells. B) Representative flow showing IFNg production and CD107 expression of CD4CAR or D1D2CAR+ CD8 T cells after stimulation with Env- target cells (CEM), Env+ target cells (activated ACH2s) or IL16 *ex vivo*. C) Summary of CAR CD8 T cells ex vivo stimulation. * p<0.01, **p<0.001, ***p<0.0001 by Mann-Whitney test.

To examine whether the D1D2CAR expressing cells derived from HSCs can suppress HIV-1 replication *in vivo*, humanized BLT mice transplanted with unmodified HSCs or HSCs modified with CD4CAR or D1D2CAR were infected with an R5-tropic HIV_NFNSXSL9_ for 10 weeks. Plasma viral load was monitored bi-weekly after HIV-1 infection. Mice that received HSCs transduced with either the CD4CAR or the D1D2CAR vectors showed lower levels of viral load for 10 weeks compared to untreated control animals (1 log lower than unmodified control mice) **(Fig 4A).** To determine if CAR T cells (defined as CD45+CD2+CD56-GFP+ to include CD3-/TCR- CAR T cells for the rest of the study), can respond to Env+ target cells or IL-16 nonspecific stimulation *ex vivo*, splenocytes from CD4CAR or D1D2CAR mice were exposed to Env+ ACH2 cells, Env- parental CEM cells, or soluble IL-16 cytokine. Afterwards, cells were analyzed for intracellular expression of IFN-γ and the degranulation marker CD107a. Full length CD4CAR T cells responded to both Env+ and IL-16 cytokine stimulation, suggesting non-specific signaling via IL-16 mediated CD4 dimerization **(Fig 4B and 4C)**. In contrast, D1D2CAR T cells responded only to Env expressing target cells, but not to soluble IL-16. These results suggest that relative to the full length CD4CAR, D1D2CAR has comparable function, does not mediate HIV-1 infection or IL-16 signaling, and has a favorable expression pattern on T cells *in vivo*.

### Costimulatory domain 4-1BB, but not CD28, allows for successful CAR T cell development from HSCs

**Fig 5:**
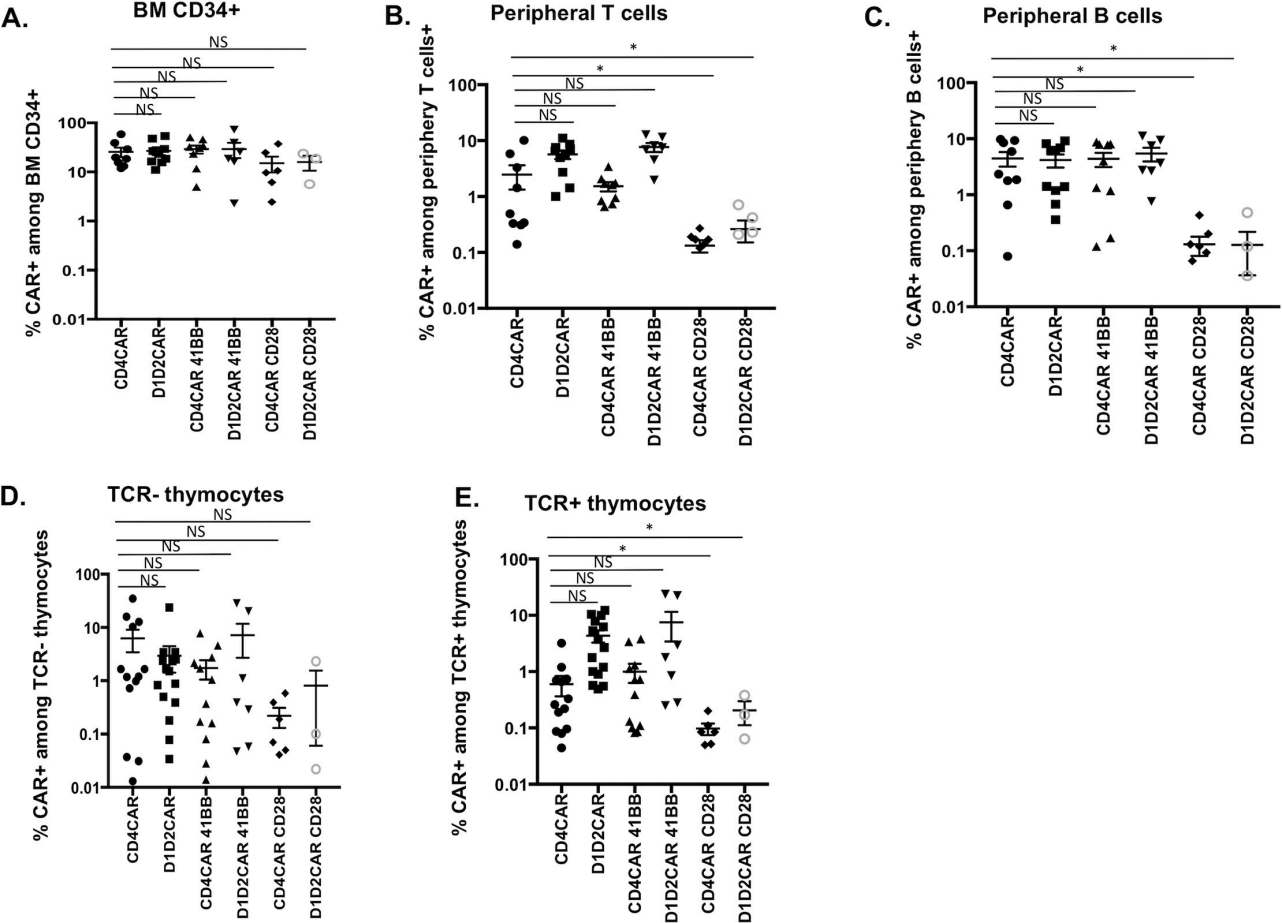
Costimulatory domain 4-1BB, but not CD28, allows for successful CAR T cell development. Humanized BLT mice were transplanted with CD34+ cells transduced with either CD4CAR, D1D2CAR, CD4CAR 4-1BB, D1D2CAR 4-1BB, CD4CAR CD28 or D1D2CAR CD28 lentiviruses. **A)** CAR+% among CD34+ hematopoietic stem cells from bone marrow 20 weeks after engraftment. **B)** CAR+% among peripheral T cell (CD45+CD2+CD56-). **C)** CAR+% among peripheral B cell. **D)** CAR+% among CD5+CD2+TCR- thymocytes. **E)** CAR+% among CD5+CD2+TCR+ thymocytes. * p<0.01, **p<0.001, ***p<0.0001 by Mann-Whitney test.

We have demonstrated that HSCs modified with a CD4CAR can differentiate into naïve CAR T cells^36^. Similar to primary responses of endogenous naïve T cells, our previous study suggested that upon first contact with antigen (HIV-1 env/SHIV-1 infected cells), these cells are delayed in their ability to become fully activated and functional [13, 15, 36]. Addition of co-stimulatory domains to the CAR molecule may facilitate more rapid primary responses of CAR+ cells, but their impact on HSC engraftment and thymopoiesis is unknown. To determine whether the costimulatory domains 4-1BB or CD28 enhance *in vivo* functions of CAR T cells, we constructed CD4CAR or D1D2CAR molecules (in a lentiviral vector containing CCR5 shRNA and sh516, as shown in **Fig 2A**) with either the 4-1BB or CD28 costimulatory domains (see **S1 Fig** for the schematic representation of the lentiviral plasmids). Humanized BLT mice were transplanted with donor matched fetal thymus and liver derived HSC (CD34+) transduced with either CD4CAR, D1D2CAR, CD4CAR-41BB, D1D2CAR-41BB, CD4CAR-CD28 or D1D2CAR-CD28. Transduction efficiency of CD34+ by these vectors was evaluated following *in vitro* culture of CD34+ cells and the levels did not appear to correlate to observed qualitative or quantitative differences between groups (**S2 Fig**). 20 weeks after transplantation, we examined HSC engraftment in the bone marrow and found that the percentage of CAR-expressing cells among CD34+ HSC is similar across different CARs, suggesting successful engraftment of all gene modified HSC **(Fig 5A)**. However, addition of the CD28 co-stimulatory domain appeared to have detrimental effect on T cell and B cell differentiation: we observed significantly reduced levels of mature CAR T **(Fig 5B)** or CAR B cells **(Fig 5C)** in peripheral blood for CD4CAR-CD28 and D1D2CAR-CD28 as compared to mice that were transplanted with CD4CAR, D1D2CAR, CD4CAR 4-1BB and D1D2CAR 4-1BB modified HSCs. Interestingly, both CD4CAR-CD28 and D1D2CAR-CD28 have significantly lower level of CAR+ cells among both TCR+ and TCR- thymocytes as compared to CARs without costimulatory molecule or with 4-1BB **(Fig 5D–5E)**, suggesting that the impairment of CAR T cell development by addition of CD28 happened prior to TCR rearrangement. Notably, 4-1BB does not affect T cell or B cell differentiation of either CD4CAR or D1D2CAR modified HSCs.

### The costimulatory domain 4-1BB enhances CAR T cell’s function *in vivo*

**Fig 6:**
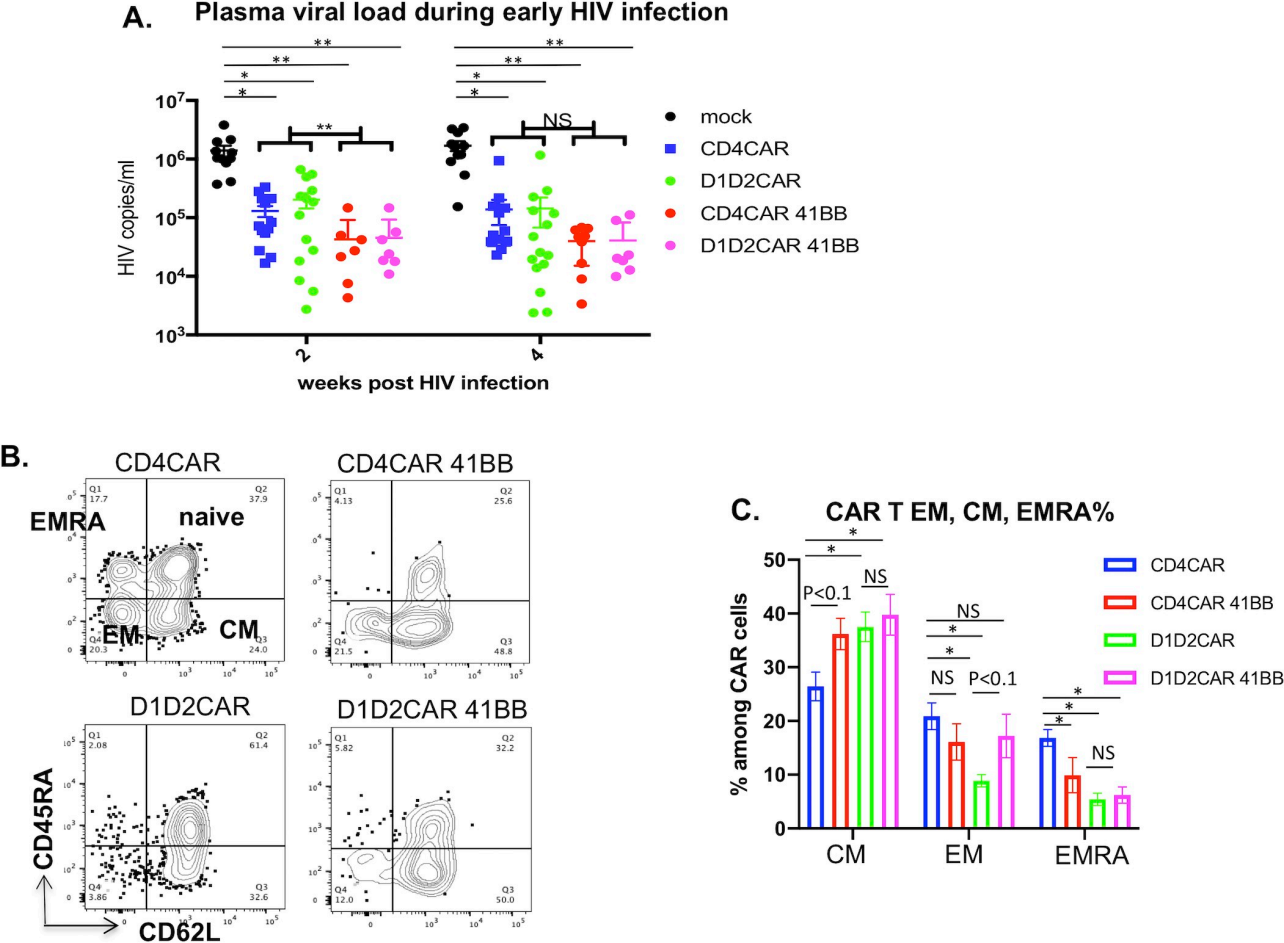
Costimulatory domain 4-1BB enhances CD4CAR T cells function. Humanized BLT mice were transplanted with control, CD4CAR, D1D2CAR, CD4CAR 4-1BB or D1D2CAR 4-1BB lentivirus transduced CD34+ cells and infected with HIV-1 after immune reconstitution**. A)** Plasma viral load 2 and 4 weeks during untreated acute HIV infection. **B)** Naïve, effector memory (EM), central memory (CM) and terminally differentiated effector memory (EMRA)% of CAR T cells (CD45+CD2+CD56-) 4 weeks after HIV infection. C) Summary of % EM, effector EMRA among CAR T cells 4 weeks after infection. * p<0.01, **p<0.001, ***p<0.0001 by Mann-Whitney test.

To examine if the costimulatory molecule 4-1BB improves CAR T cell function in vivo, we infected humanized BLT mice transplanted with untransduced HSCs, or HSCs transduced with protective CD4CAR, D1D2CAR, CD4CAR-41BB or D1D2CAR-41BB with R5-tropic HIV_NFNSXSL9_ after immune reconstitution. All of the CAR mice showed significant suppression of viral load as compared to control mice **(Fig 6A)** at 2 and 4 weeks after HIV-1 infection. Mice that received CD4CAR-41BB and D1D2CAR-41BB HSPC transplants demonstrated significantly lower viral loads than mice that received CD4CAR and D1D2CAR HSC transplants during early infection (week 2), suggesting that the costimulatory domain triggers a faster and more effective response. In peripheral CAR T cell based therapies, co-stimulatory signals are required to achieve persistence and robust anti-tumor/anti-HIV-1 activity [14, 37]. To examine how the 4-1BB costimulatory domain affects CAR T cell differentiation into naïve, effector, memory and terminally differentiated effector memory T cells in vivo in response to antigen stimulation, we performed flow cytometry on peripheral PBMCs collected 4 weeks after HIV-1 infection. We found that compared to CD4CAR, CD4CAR 4-1BB CAR T had increased central memory (CM) differentiation (p<0.1) and significantly reduced terminally differentiated effector memory cells (EMRA), similar to what was previously reported for CD19CAR T cells [37] **(Fig 6B and 6C)**. Interestingly, we found that the D1D2CAR had reduced effector memory (EM) differentiation as compared to the CD4CAR **(Fig 6C)**. D1D2CAR 4-1BB had increased EM differentiation as compared to D1D2CAR, to the level similar to CD4CAR and CD4CAR 4-1BB. Importantly, CD4CAR-41BB, D1D2CAR 4-1BB and D1D2CAR T cells all have significantly reduced EMRA differentiation, which are terminally differentiated T cells that display senescent phenotypes and are thus less desirable [38] **(Fig 6C)**. While EM differentiation is critical in antiviral responses, the skewing of the 4-1BB containing CD4CAR and D1D2CAR towards central memory type responses is a desirable aspect of the response as central memory (CM) T cells have superior persistence and proliferation and can support sustained response for longer periods and maintain immune memory [39]. In comparison to 4-1BB containing CARs, CD28-containing CAR BLT mice showed no expansion of CAR T cells after 8 weeks untreated HIV challenge (**S3A Fig**) and did not suppress viral replication (**S3B Fig**).

### D1D2CAR 4-1BB cells show faster viral suppression in combination with ART and better CAR T cell persistence during ART treatment

**Fig 7:**
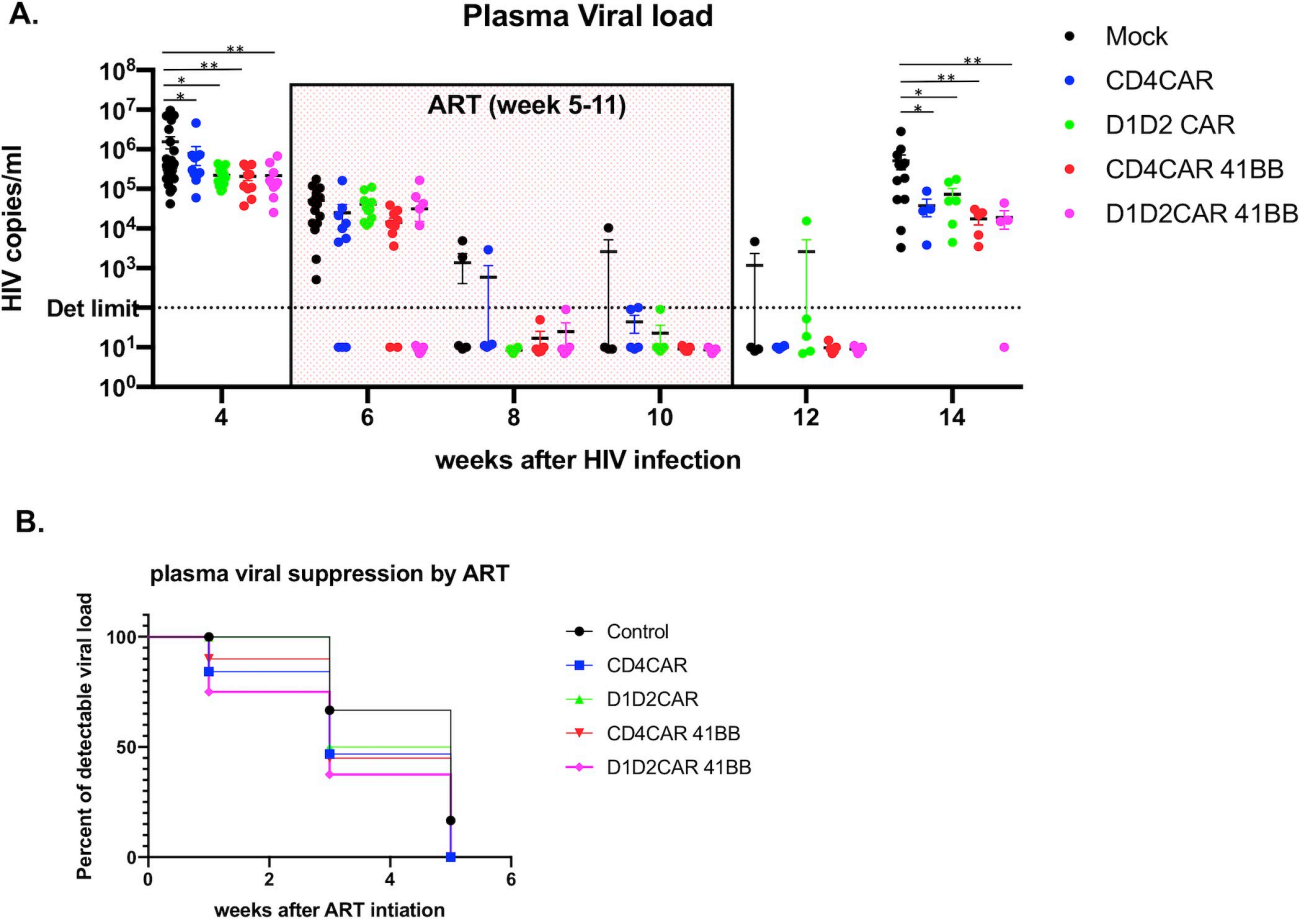
D1D2CAR 4-1BB CAR T cells show faster viral suppression in combination with ART. Humanized BLT mice were transplanted with control, CD4CAR, D1D2CAR, CD4CAR 4-1BB or D1D2CAR 4-1BB lentivirus transduced CD34+ cells and infected with HIV-1 after immune reconstitution. 4 weeks after HIV infection, mice were treated with ART from week 5–11, followed by ART interruption. **A)** Plasma viral load over time. * p<0.01, **p<0.001, ***p<0.0001 by Mann-Whitney test. **B)** Survival analysis of time to undetectable viral load for each group.

To examine the effects of CD4-CAR HSC-based therapy on HIV-1 suppression, persistence, and viral rebound, we constructed BLT mice with HSCs modified with CD4CAR, CD4CAR 4-1BB, D1D2CAR and D1D2CAR 4-1BB containing lentiviral vectors and infected with R-5 tropic HIV_NFNSXSL9_ after immune reconstitution. 5 weeks after HIV-1 infection, mice were put on daily ART (TDF, FTC and Elvitegravir) for 6 weeks (allowing for full viral suppression) followed by ART withdrawal. Plasma viral loads were measured bi-weekly prior to, during, and after ART **(Fig 7A)**. With the exception of one mouse in the mock group, we observed full viral suppression by ART to undetectable levels in each mouse by week 11 **(Fig 7A)**. Survival analysis of time to undetectable viral loads shows that D1D2CAR-41BB mice had significantly faster viral suppression as compared to mock mice (Log rank test, p = 0.0375) **(Fig 7B)**. 3 weeks following ART release, all mice rebounded except one mouse in D1D2CAR 4-1BB group. All CAR-expressing mice maintained lower viral load as compared to mock mice after viral rebound (Fig 7A) at a point lower than pre ART levels. D1D2CAR-41BB mice exhibited lower average viral loads as compared to D1D2CAR but did not reach statistical significance.

**Fig 8:**
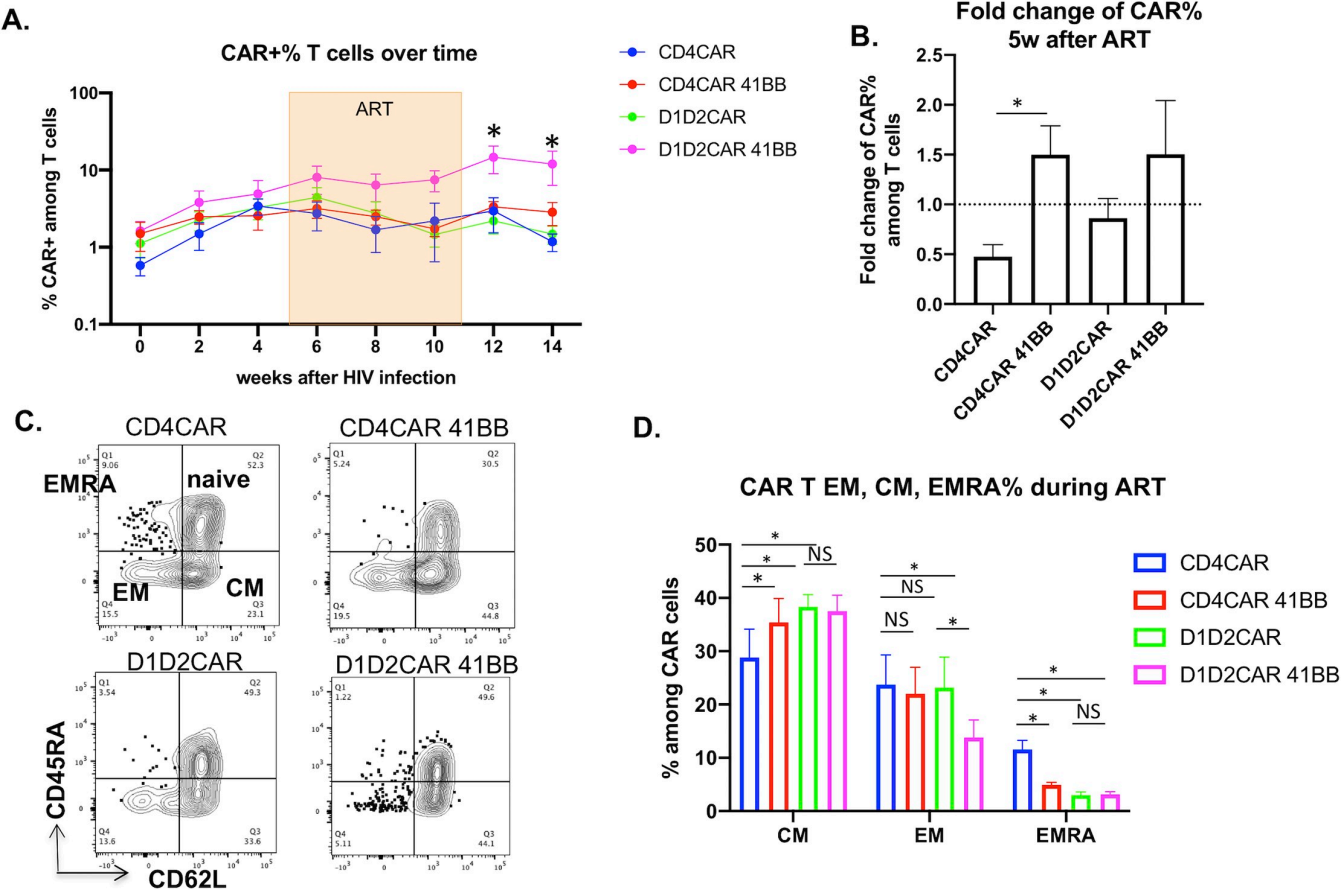
CD4CAR 4-1BB T cells and D1D2CAR 4-1BB CAR T cells show better persistence during ART treatment. **A)** summary of %CAR+ among T cells (CD45+CD2+CD56-) in peripheral blood of humanized BLT mice. **B)** Fold change in CAR+% among T cells 5 weeks after ART (week 10 after HIV infection) as compared to before ART treatment (week 4 after HIV infection). **C)** Naïve, effector memory (EM), central memory (CM) and terminally differentiated effector memory (EMRA)% of CAR T cells 5 weeks after ART treatment (10 weeks after HIV infection). **D**) Summary of % EM, effector EMRA among CAR T cells 3 weeks after ART treatment weeks after ART treatment. * p<0.01, **p<0.001, ***p<0.0001 by Mann-Whitney test.

We then closely examined the change of CAR+ T cells over time **(Fig 8)**. As expected, all CAR T cells expanded after HIV-1 infection **(Fig 8A)**. Interestingly, during ART treatment, numbers of CAR+ T cells in both CD4CAR and D1D2CAR containing mice declined after ART, likely due to reduction of viral antigen and consequent stimulation. Interestingly, percentages of CAR+ T cells in CD4CAR 4-1BB and D1D2CAR 4-1BB containing mice were maintained in the peripheral blood during ART treatment **(Fig 8B)**. After ART withdrawal, D1D2CAR 4-1BB CAR T cells further expanded and had significantly higher percentages of CAR+ T cells in the peripheral blood as compared to other groups **(Fig 8A).** CM populations of CAR+ T cells developed in each CAR-expressing group of animals, albeit to different degrees **(Fig 8C–8D)**. Compared to CD4CAR, CD4CAR 4-1BB, D1D2CAR and D1D2CAR 4-1BB mice had significantly higher percentages of CM CAR T cells and lower percentages of EMRA CAR T cells (**Fig 8D**). Interestingly, while D1D2CAR 4-1BB mice had higher percentages of EM phenotype during early HIV-1 infection as compared to D1D2CAR mice (**Fig 6C**), during ART viral suppression, D1D2CAR 4-1BB mice had lower level of EM CAR T phenotype as compared to D1D2CAR mice (**Fig 8D**). This strongly suggests a skewing of D1D2CAR 41BB to develop into CM phenotype during ART mediated viral suppression, which may explain the better persistence of D1D2CAR 4-1BB T cells during ART treatment when HIV-1 viral antigen is scarce.

## Discussion

Recent years have seen rapid developments in CAR-T therapy. Our study further contributes a superior CD4CAR-based molecule, D1D2CAR-4-1BB, that is suitable for hematopoietic stem cell based CAR therapy and can achieve potent, long lasting anti-viral responses *in vivo*. Numerous studies have shown that the second and third generation of CAR molecules that incorporate the signaling domains of co-stimulatory molecules such as CD28 or 4-1BB into the CAR molecule demonstrate enhanced CAR T cell function and survival [40]. For anti-HIV-1 CARs, recent studies showed that both CD28 and 4-1BB enhanced the anti-HIV-1 function of CD4-based CAR-transduced peripheral T cells *in vitro* [14], and a 4-1BB containing CAR allowed better expansion and control of HIV-1 spread in vivo [14, 41]. However, most anti-HIV-1 CAR studies focused on peripheral based T cells and their short term effects on HIV-1 infection in vivo [14, 41–43]. It is unclear if this peripheral anti-HIV-1 CAR T therapy can achieve long term persistence.

For successful long-term immune containment of HIV-1 infection, it is critical to have transduced T cells that normally survive and proliferate *in vivo* over decades, to suppress viral reactivations from the long-lived latent reservoir. Previously, using a CD4-based first generation CAR without a co-stimulatory domain, we demonstrated the feasibility and efficacy of using HSC based CAR therapy to achieve lifelong engraftment, development, and persistence of functional CAR T cells that trafficked to multiple lymphoid tissues and suppressed viral replication *in vivo* [13, 16, 36]. To further enhance the safety and efficacy of HSC-based CAR therapy, here we tested a novel, truncated D1D2CAR designed to eliminate IL-16 mediated signaling and CD4CAR mediated HIV-1 infection. We also tested the addition of co-stimulatory domains CD28 and 4-1BB. We found that D1D2CAR did not mediate HIV-1 infection (**Fig 1B**), did not react to IL-16 stimulation (**Fig 4B and 4C**) and had similar anti-HIV-1 activity as compared to CD4CAR both in vitro and in vivo (**Figs 2B, 2C and 4A**). Interestingly, unlike CD4CAR, which binds to MHC-II and affects TCR gene arrangement and T cell differentiation, we found that D1D2CAR had no effect on T cell differentiation and TCR expression (**Fig 3B–3D**). As a result, while CD4CAR modified HSCs had biased development towards CAR B cells, D1D2CAR HSCs had unbiased development of T and B lymphocytes (**Fig 3B**). Importantly, we found that while the 4-1BB costimulatory domain did not affect CAR T cell development for both CD4CAR and D1D2CAR, addition of CD28 severely impacted T cell and B cell development (**Fig 5B–5E**), suggesting that adding the CD28 co-stimulatory domain is deleterious for HSC based CAR therapies. As CAR therapy advances, third or fourth generations of CAR designs have emerged by combining multiple co-stimulatory domains and/or inclusion of cytokine receptor stimulatory domains for IL-7, IL12, or IL-15 to improve CAR T cell expansion and persistence [44, 45]. However, incorporating additional co-stimulatory domains may affect thymus-mediated T cell differentiation. Therefore, the effects of additional signaling domains and cytokines on HSC-based CAR therapy would need to be tested in the context of each individual CAR molecule.

Recent studies suggest that incorporation of 4-1BB in certain CARs could improve memory formation and ameliorate exhaustion [21, 26, 46]. However, the clinical impact of choosing and combining different costimulatory domains for different CAR molecules and diseases has yet to be clearly established^38^. In our study, we found that addition of the 4-1BB costimulatory domain resulted in faster viral suppression in CD4CAR 4-1BB and D1D2CAR 4-1BB mice as compared to CD4CAR and D1D2CAR mice (**Fig 6A** without ART, **Fig 7A and 7B** with ART). 4-1BB also promoted better CAR T cell persistence during ART treatment when viral antigen was drastically reduced (**Fig 8A and 8B**), particularly for D1D2CAR 4-1BB T cells. In general, we observed skewing of CM and reduction in EMRA cells for CD4CAR-41BB, D1D2CAR 4-1BB, and D1D2CAR 4-1BB T cells during early infection and during ART treatment. Compared to CD4CAR, D1D2CAR T cells have reduced EM formation, improved formation of CM cells and reduction in EMRA cells, potentially due to reduction in nonspecific signaling via HLA class II and IL-16. Interestingly, the impact of 4-1BB on D1D2CAR T cells versus on CD4CAR T cells differ slightly: CD4CAR 4-1BB T cells have improved CM formation and reduced formation of EMRA as compared to CD4CAR T cells during untreated primary infection and ART treatment; in contrast, D1D2CAR 4-1BB T cells showed increased EM differentiation as compared to D1D2CAR T cells during untreated primary HIV-1 infection and reduced EM phenotype during ART treatment. 4-1BB may have promoted naïve to EM differentiation for D1D2CAR 4-1BB T cells during early infection and promoted transition of D1D2CAR 4-1BB T cells from EM to CM during ART infection. The detailed mechanism of 4-1BB impact on CD4CAR and D1D2CAR T cells is yet to be studied. The fact that 4-1BB has differential impact on two closely related CD4-based CARs strongly suggests that the utilization and selection of costimulatory domains should be carefully selected based on the context of the CAR molecule and the disease.

To achieve ART-free remission or “HIV-1 cure”, it is crucial to have long term anti-HIV-1 immunity to control reactivation of latently infected cells. The critical role of HIV-1 Env interaction with CD4 for viral replication limit potential viral escape from a CD4-based CAR, since loss of Env binding to CD4 is associated with marked loss of viral fitness [47].Our HLA-independent, CD4 based HSC-derived CAR therapy could provide a long term, enhanced anti-HIV-1 immunity that is also resistant to HIV-1 immune evasion. Given the persistence and functionality of CD4 based CAR T cells derived from HSCs, our HSCs based CAR therapy could also be combined with multiple rounds of latency reversal agents as an effective ‘kill’ part for the ‘kick-and-kill’ strategy to reduce and eliminate HIV-1 reservoir [48].

## Materials and methods

### Ethics statement

This study was carried out in strict accordance with the recommendations in the Guide for the Care and Use of Laboratory Animals of the National Institutes of Health (”The Guide”), and was approved by the Institutional Animal Care and Use Committees of the University of California, Los Angeles, protocol #2010-038-31J. For humanized mice, all surgeries were performed under ketamine/xylazine and isofluorane anesthesia and all efforts were made to minimize animal pain and discomfort.

#### Humanized mice construction

CAR BLT mice were constructed similarly to previously reported HIV-1 Triple CAR BLT humanized mice (1, 2). Briefly, human fetal liver derived CD34+ cells were purified by immunomagnetic separation. Cells were then transduced overnight with control or protective CAR lentiviruses with retronectin-coated plates. To evaluate transduction efficiency, 0.1 million transduced CD34+ cells was set aside from surgery and were cultured in extension media (IL-3 100 ng/ml, IL-6 100 ng/ml, SCF 100 ng/ml in 10% FCS RPMI 1640) for 7 days. Afterwards, cells were analyzed by flow cytometry. On day of transplant, NOD.Cg-PrkdcscidIl2rgtm1Wjl/SZJ (NOD/SCID/IL2Rγ−/− or NSG; The Jackson Laboratory) mice received 2.7 Gy total body sublethal irradiation and then were transplanted with transduced CD34+ in Matrigel (corning Life Sciences), liver and thymus tissue under the kidney capsule same donor as the CD34+ cells. Afterward, mice were injected with ∼0.5×10^6^ lentivirus-based CAR vector transduced CD34+ cells. At 8–10 weeks post-transplantation, each mouse was bled retro-orbitally and peripheral blood mononuclear cells analyzed by flow cytometry to check human immune cell engraftment. Upon stable human leukocyte reconstitution efficiency more than 50%, mice were used for HIV-1 infection and further experiments. To supplement for low level of dendritic cells in NSG-BLT mice [13], all mice were injected with 1 million unmodified dendritic cells cultured from autologous HSCs (prepared as detailed in [49]) prior to HIV infection or before ART withdrawal.

#### Lentiviral production

The lentivirus based GFP control vector and CAR vectors were produced in 293FT cells using the Lipofectamine 2000 reagent (Invitrogen, Carlsbad, CA) as previously described[13]. Briefly, 293FT cells were co-transfected simultaneously with CAR vectors with pCMV.ΔR8.2.Δvpr packaging construct and the pCMV-VSV-G envelope protein plasmid as previously described. Supernatant was collected from transfected 293FT cells 48 hours following transfection, filtered using a 0.45 *μm* sterile filter, and concentrated by ultracentrifugation using a Beckman SW32 rotor at 30,000 rpm at 4°C. Medium was aspirated and pellet was resuspended with PBS and stored at -80°C.

#### Cytokine assay

Primary CD8 T cells were purified from healthy PBMCs using CD8 microbeads (Miltenyi #130-097-057). Afterwards, CD8 cells were stimulated with plate bound anti-CD3 and soluble anti-CD28 antibodies for 2 days and transduced with protective CD4CAR or D1D2CAR lentiviral vector for 2 days. Infected T1 cells were prepared by infection of T1 cells with 500ng p24 of HIV_NL4-3_/million cells for 2 days. CAR T cells were co-incubated at with either uninfected or HIV-1 infected T1 cells at 1:10 (Effector: Target) ratio for 16 hours (overnight). Afterwards, cells were treated with GolgiPlug for 6hours before intracellular staining of cytokines and flow cytometry analysis.

#### CTL assay

Primary CD8 T cells were purified from healthy PBMCs using CD8 microbeads (Miltenyi #130-097-057). Afterwards, CD8 cells were stimulated with plate bound anti-CD3 and soluble anti-CD28 antibodies for 2 days and transduced with protective CD4CAR or D1D2CAR lentiviral vector for 2 days. Target HIV Env expressing (Env+) cells or Env- control cells were prepared by stimulating HIV latently infected U1 cells overnight with PMA and ionomycin or mock stimulation. Prior to CTL assay, target cells were washed and pre-stained with Celltrace Farred (ThermoFisher #C34564). Afterwards, CD8 CAR T cells were coincubated with stimulated (Env+) or unstimulated (Env-) U1 cells at 1:1, 3:1, 5:1 effector to target ratio for 16 hours. For control, Env+ or Env- U1 cells cells were cultured alone without CAR cells at similar condition. Afterwards, cells were stained with zombie violet fixable viability dye (Biolegend #423113), fixed and stained intracellularly using BD Cytofix/Cytoperm kit (BD #554714) for HIV core antigen (Clone KC57, Beckman Coulter, #6604667) and analyzed by flow cytometry using a MACSQuant analyzer 10 (Miltenyi) and flowjo (BD). % specific killing was calculated by (%live gag+ U1 cells without CAR cell—%live gag+ U1 cells with CAR cells)/ %live gag+ U1 cells without CAR cells.

#### HIV-1 infection and ART treatment

The R5 tropic strain of HIV-1 (NFNSXSL9) was generated by transfection of 293T cells with plasmid containing full-length HIV-1 (NFNSXSL9) genome. Humanized mice were infected with NFNSXSL9 (200 ng p24 per mouse) through retro-orbital injection while under inhalant general anesthesia. Infected mice with demonstrable viral infection were treated for 6 weeks with ART drugs. The ART regimen is consisted of tenofovir disproxil-fumarate (TDF, 80mg/kg), emtricitabine (FTC, 120mg/kg), and Elvitegravir (ELV160mg/kg) given by food. TDF, FTC and ELV were generously supplied by Gilead Sciences. TDF, FTC and ELV were dissolved in DMSO and mixed with sweetened moist gel meal (DietGel Boost, ClearH2O; Medidrop Sucralose).

#### Mouse blood and tissue collection

Peripheral blood samples were collected at approximately 12 weeks of age using retro-orbital bleeding. Red blood cells were lysed using Red Cell lysis buffer solution (Sigma-Aldrich), and the remaining cells were washed with PBS. This was followed by further staining for flow cytometry analyses. Tissue samples were collected on MACS tissue storage solution (130-100-008) at necropsy and processed immediately for single cell isolation and flow cytometry analysis. After debris removal, cell suspension was filtered with sterile 70 μm filter (Fisher Scientific) and processed by staining or DNA/RNA extraction.

#### Viral load assay

Blood collected with EDTA anticoagulant from retro-orbital biweekly bleeding or heart puncture during scarification. Blood was then spun at 1,200 g to collect plasma supernatant. Cell free plasma viral RNA was purified using a QIAamp Viral RNA Mini Kit (QIAGEN). HIV-1 RNA was quantified by real-time RT-PCR using TaqMan RNA-To-Ct One-Step reagents (Thermo Fisher Scientific) with primers HIV-1_F: 50-CAATGGCAGCAATTTCACCA-30 and HIV-1_R: 50-GAATGC CAAATTCCTGCTTGA-30 and a probe hybridizing to HIV01 NL4-3 HIV-1 probe: 5′-[6-FAM]CCCACCAACAGGCGGCCTTAACTG[Tamra-Q]-3′[13].

#### Flow cytometry

For surface staining, single-cell suspensions prepared from peripheral blood, spleen, brain or bone marrow of humanized mice were stained with surface markers and acquired on a LSRFortessa flow cytometer and FACSDiva software (BD Biosciences). For intracellular staining, cells were first stained with surface markers, and then fixed and permeabilized with Cytofix/Cytoperm buffer (BD Biosciences), followed by intracellular staining. The following antibodies were used in flow cytometry: CD45 (clone HI30), CD2(clone RPA-2.10), CD8 (clone SK1), CD14 (clone 61D3), CD19 (clone HIB19), PD-1 (clone ebioJ105) (all above from BD Biosciences), CD3 (clone OKT3), CD4 (clone RPA-T4), CD45RA (clone H100), CD62L (clone DREG-56), CD38 (clone HIT2), HLA-DR (clone L240), TIM-3 (clone F38-2E2), IFN-γ (clone 4S.B3), TNF-α (clone MAb11), CD107a (clone eBioH4A3) (all above from Biolegend), TCR Vβ17(clone E17.5F3.15.13) (Beckman Coulter). LIVE/DEAD Fixable Yellow Dead Cell Stain Kit were purchased from Invitrogen. All antibody used for staining were human specific and fluorochrome conjugated to BV605, PE, PerCP-Cy5.5, PE-Cy5, PE-Cy7, ECD, APC, APC–eFluor 780, Alexa Fluor 700, eFluor 405, BV510, or Pacific Blue in an appropriate combination. Data were analyzed using FlowJo software.

### Statistical analysis

Statistical analysis was performed using software Prism. Man-Whitney U test is used for nonparametric testing of independent groups. Wilcoxon matched pairs signed rank test was used for nonparametric testing of paired groups. Log rank test was used for survival analysis.

## Supporting information

S1 Fig Schematic review of protective CD4CAR, D1D2CAR, CD4CAR 4-1BB, D1D2CAR 4-1BB, CD4CAR CD28 and D1D2CAR CD28 lentiviruses. (TIFF)

S2 Fig CD34+ transduction efficiency of CAR humanized BLT mice. Humanized mice were constructed with donor matched fetal thymus and liver derived CD34+ cells transduced with either CD4CAR, D1D2CAR, CD4CAR 4-1BB, D1D2CAR 4-1BB, CD4CAR CD28 or D1D2CAR CD28. 0.1 million transduced CD34+ cells were set aside from transplant and were cultured in extension culture for 7 days. Afterwards, cells were analyzed by flow cytometry.
(TIFF)

S3 Fig CD28 containing CAR mice shows no expansion of CAR+ T cells or suppression of HIV replication. Humanized BLT mice were transplanted with either mock transduced CD34+, or CD34+ cells transduced with lentiviruses CD4CAR CD28 or D1D2CAR CD28. Mice were challenged with HIVNFN_SXSL9_ after immune constitution. A) GFP+CAR+% among T cells (CD45+CD2+CD56-) were measured before infection and 8 weeks after infection. B) plasma viral load was measured 8 weeks post infection. * p<0.01, **p<0.001, ***p<0.0001 by Mann-Whitney test.
(TIFF)

## References

[1] Siliciano R. F. & Greene W. C. HIV latency. Cold Spring Harbor perspectives in medicine 1, a007096, 10.1101/cshperspect.a007096 (2011). 22229121 PMC3234450

[2] Margolis D. M., Garcia J. V., Hazuda D. J. & Haynes B. F. Latency reversal and viral clearance to cure HIV-1. *Science* 353, aaf6517, 10.1126/science.aaf6517 (2016). 27463679 PMC5021637

[3] Rasmussen T. A. et al. Panobinostat, a histone deacetylase inhibitor, for latent-virus reactivation in HIV-infected patients on suppressive antiretroviral therapy: a phase 1/2, single group, clinical trial. *Lancet HIV* 1, e13–21, 10.1016/S2352-3018(14)70014-1 (2014). 26423811

[4] Gupta R. K. et al. HIV-1 remission following CCR5Delta32/Delta32 haematopoietic stem-cell transplantation. *Nature* 568, 244–248, 10.1038/s41586-019-1027-4 (2019). 30836379 PMC7275870

[5] Hutter G. et al. Long-term control of HIV by CCR5 Delta32/Delta32 stem-cell transplantation. *The New England journal of medicine* 360, 692–698, 10.1056/NEJMoa0802905 (2009). 19213682

[6] The Lancet H. Like London buses, two putative cure cases arrive at once. *Lancet HIV* 6, e205, 10.1016/S2352-3018(19)30086-4 (2019). 30942184

[7] Hutter G. More on shift of HIV tropism in stem-cell transplantation with CCR5 delta32/delta32 mutation. *The New England journal of medicine* 371, 2437–2438, 10.1056/NEJMc1412279 (2014). 25517721

[8] Kordelas L. et al. Shift of HIV tropism in stem-cell transplantation with CCR5 Delta32 mutation. *The New England journal of medicine* 371, 880–882, 10.1056/NEJMc1405805 (2014). 25162903

[9] Yang O. O. et al. Lysis of HIV-1-infected cells and inhibition of viral replication by universal receptor T cells. *Proceedings of the National Academy of Sciences of the United States of America* 94, 11478–11483, 10.1073/pnas.94.21.11478 (1997). 9326635 PMC23511

[10] Deeks S. G. et al. A phase II randomized study of HIV-specific T-cell gene therapy in subjects with undetectable plasma viremia on combination antiretroviral therapy. *Molecular therapy*: *the journal of the American Society of Gene Therapy* 5, 788–797, 10.1006/mthe.2002.0611 (2002). 12027564

[11] Mitsuyasu R. T. et al. Prolonged survival and tissue trafficking following adoptive transfer of CD4zeta gene-modified autologous CD4(+) and CD8(+) T cells in human immunodeficiency virus-infected subjects. *Blood* 96, 785–793 (2000). 10910888

[12] Scholler J. et al. Decade-long safety and function of retroviral-modified chimeric antigen receptor T cells. *Science translational medicine* 4, 132ra153, 10.1126/scitranslmed.3003761 (2012). 22553251 PMC4368443

[13] Zhen A. et al. HIV-specific Immunity Derived From Chimeric Antigen Receptor-engineered Stem Cells. *Molecular therapy*: *the journal of the American Society of Gene Therapy* 23, 1358–1367, 10.1038/mt.2015.102 (2015). 26050990 PMC4817874

[14] Leibman R. S. et al. Supraphysiologic control over HIV-1 replication mediated by CD8 T cells expressing a re-engineered CD4-based chimeric antigen receptor. *PLoS pathogens* 13, e1006613, 10.1371/journal.ppat.1006613 (2017). 29023549 PMC5638568

[15] Liu L. et al. Novel CD4-Based Bispecific Chimeric Antigen Receptor Designed for Enhanced Anti-HIV Potency and Absence of HIV Entry Receptor Activity. *Journal of virology* 89, 6685–6694, 10.1128/JVI.00474-15 (2015). 25878112 PMC4468509

[16] Zhen A. et al. Long-term persistence and function of hematopoietic stem cell-derived chimeric antigen receptor T cells in a nonhuman primate model of HIV/AIDS. *PLoS pathogens* 13, e1006753, 10.1371/journal.ppat.1006753 (2017). 29284044 PMC5746250

[17] Rafiq S., Hackett C. S. & Brentjens R. J. Engineering strategies to overcome the current roadblocks in CAR T cell therapy. *Nat Rev Clin Oncol* 17, 147–167, 10.1038/s41571-019-0297-y (2020). 31848460 PMC7223338

[18] Zhen A. et al. Stem-cell Based Engineered Immunity Against HIV Infection in the Humanized Mouse Model. *Journal of visualized experiments*: *JoVE*, 10.3791/54048 (2016). 27404517 PMC4993349

[19] Vatakis D. N. et al. Introduction of exogenous T-cell receptors into human hematopoietic progenitors results in exclusion of endogenous T-cell receptor expression. *Molecular therapy*: *the journal of the American Society of Gene Therapy* 21, 1055–1063, 10.1038/mt.2013.28 (2013). 23481324 PMC3666627

[20] Kitchen S. G. et al. In vivo suppression of HIV by antigen specific T cells derived from engineered hematopoietic stem cells. *PLoS pathogens* 8, e1002649, 10.1371/journal.ppat.1002649 (2012). 22511873 PMC3325196

[21] Long A. H. et al. 4-1BB costimulation ameliorates T cell exhaustion induced by tonic signaling of chimeric antigen receptors. *Nature medicine* 21, 581–590, 10.1038/nm.3838 (2015). 25939063 PMC4458184

[22] Savoldo B. et al. in *The Journal of clinical investigation* Vol. 121 1822–1826 (2011). 21540550 10.1172/JCI46110 PMC3083795

[23] Drent E. et al. Combined CD28 and 4-1BB Costimulation Potentiates Affinity-tuned Chimeric Antigen Receptor-engineered T Cells. *Clinical cancer research*: *an official journal of the American Association for Cancer Research* 25, 4014–4025, 10.1158/1078-0432.CCR-18-2559 (2019). 30979735 PMC7477921

[24] Turtle C. J. & Maloney D. G. Clinical trials of CD19-targeted CAR-modified T cell therapy; a complex and varied landscape. *Expert Rev Hematol* 9, 719–721, 10.1080/17474086.2016.1203251 (2016). 27322438

[25] Ramos C. A., Savoldo B. & Dotti G. CD19-CAR trials. *Cancer journal (Sudbury*, *Mass*.*)* 20, 112–118, 10.1097/PPO.0000000000000031 (2014). 24667955 PMC3979594

[26] Kawalekar O. U. et al. Distinct Signaling of Coreceptors Regulates Specific Metabolism Pathways and Impacts Memory Development in CAR T Cells. *Immunity* 44, 380–390, 10.1016/j.immuni.2016.01.021 (2016). 26885860 PMC13498396

[27] Imai C. et al. Chimeric receptors with 4-1BB signaling capacity provoke potent cytotoxicity against acute lymphoblastic leukemia. *Leukemia* 18, 676–684, 10.1038/sj.leu.2403302 (2004). 14961035

[28] Zhen A. et al. Correction: Long-term persistence and function of hematopoietic stem cell-derived chimeric antigen receptor T cells in a nonhuman primate model of HIV/AIDS. *PLoS pathogens* 14, e1006891, 10.1371/journal.ppat.1006891 (2018). 29529058 PMC5846784

[29] Yin Y., Wang X. X. & Mariuzza R. A. Crystal structure of a complete ternary complex of T-cell receptor, peptide-MHC, and CD4. *Proceedings of the National Academy of Sciences of the United States of America* 109, 5405–5410, 10.1073/pnas.1118801109 (2012). 22431638 PMC3325661

[30] Kwong P. D. et al. Structure of an HIV gp120 envelope glycoprotein in complex with the CD4 receptor and a neutralizing human antibody. *Nature* 393, 648–659, 10.1038/31405 (1998). 9641677 PMC5629912

[31] Esser U. et al. Molecular function of the CD4 D1 domain in coreceptor-mediated entry by HIV type 1. *AIDS research and human retroviruses* 16, 1845–1854, 10.1089/08892220050195801 (2000). 11118070

[32] Wang J. H. et al. Crystal structure of the human CD4 N-terminal two-domain fragment complexed to a class II MHC molecule. *Proceedings of the National Academy of Sciences of the United States of America* 98, 10799–10804, 10.1073/pnas.191124098 (2001). 11535811 PMC59561

[33] Liu Y. et al. Identification of a CD4 domain required for interleukin-16 binding and lymphocyte activation. *The Journal of biological chemistry* 274, 23387–23395, 10.1074/jbc.274.33.23387 (1999). 10438516

[34] Cruikshank W. W., Kornfeld H. & Center D. M. Interleukin-16. *Journal of leukocyte biology* 67, 757–766, 10.1002/jlb.67.6.757 (2000). 10857846

[35] Vignali D. A. & Vignali K. M. Profound enhancement of T cell activation mediated by the interaction between the TCR and the D3 domain of CD4. *Journal of immunology (Baltimore*, *Md*.: *1950)* 162, 1431–1439 (1999). 9973399

[36] Zhen A., Carrillo M. A. & Kitchen S. G. Chimeric antigen receptor engineered stem cells: a novel HIV therapy. *Immunotherapy* 9, 401–410, 10.2217/imt-2016-0121 (2017). 28357916 PMC5618937

[37] Weinkove R., George P., Dasyam N. & McLellan A. D. Selecting costimulatory domains for chimeric antigen receptors: functional and clinical considerations. *Clin Transl Immunology* 8, e1049, 10.1002/cti2.1049 (2019). 31110702 PMC6511336

[38] Callender L. A. et al. Human CD8(+) EMRA T cells display a senescence-associated secretory phenotype regulated by p38 MAPK. *Aging Cell* 17, 10.1111/acel.12675 (2018). 29024417 PMC5770853

[39] Srivastava S. & Riddell S. R. Chimeric Antigen Receptor T Cell Therapy: Challenges to Bench-to-Bedside Efficacy. *Journal of immunology (Baltimore*, *Md*.: *1950)* 200, 459–468, 10.4049/jimmunol.1701155 (2018). 29311388 PMC5957501

[40] Sadelain M., Brentjens R. & Riviere I. The basic principles of chimeric antigen receptor design. *Cancer discovery* 3, 388–398, 10.1158/2159-8290.CD-12-0548 (2013). 23550147 PMC3667586

[41] Maldini C. R. et al. Dual CD4-based CAR T cells with distinct costimulatory domains mitigate HIV pathogenesis in vivo. *Nature medicine*, 10.1038/s41591-020-1039-5 (2020). 32868878 PMC9422086

[42] Hale M. et al. Engineering HIV-Resistant, Anti-HIV Chimeric Antigen Receptor T Cells. *Molecular therapy*: *the journal of the American Society of Gene Therapy* 25, 570–579, 10.1016/j.ymthe.2016.12.023 (2017). 28143740 PMC5363191

[43] Anthony-Gonda K. et al. Multispecific anti-HIV duoCAR-T cells display broad in vitro antiviral activity and potent in vivo elimination of HIV-infected cells in a humanized mouse model. *Science translational medicine* 11, 10.1126/scitranslmed.aav5685 (2019). 31391322 PMC7136029

[44] Hurton L. V. et al. Tethered IL-15 augments antitumor activity and promotes a stem-cell memory subset in tumor-specific T cells. *Proceedings of the National Academy of Sciences of the United States of America* 113, E7788–e7797, 10.1073/pnas.1610544113 (2016). 27849617 PMC5137758

[45] Chmielewski M., Hombach A. A. & Abken H. Of CARs and TRUCKs: chimeric antigen receptor (CAR) T cells engineered with an inducible cytokine to modulate the tumor stroma. *Immunological reviews* 257, 83–90, 10.1111/imr.12125 (2014). 24329791

[46] Guedan S. et al. Enhancing CAR T cell persistence through ICOS and 4-1BB costimulation. *JCI insight* 3, 10.1172/jci.insight.96976 (2018). 29321369 PMC5821198

[47] Louie R. H. Y., Kaczorowski K. J., Barton J. P., Chakraborty A. K. & McKay M. R. Fitness landscape of the human immunodeficiency virus envelope protein that is targeted by antibodies. *Proceedings of the National Academy of Sciences of the United States of America* 115, E564–E573, 10.1073/pnas.1717765115 (2018). 29311326 PMC5789945

[48] Kim Y., Anderson J. L. & Lewin S. R. Getting the "Kill" into "Shock and Kill": Strategies to Eliminate Latent HIV. *Cell Host Microbe* 23, 14–26, 10.1016/j.chom.2017.12.004 (2018). 29324227 PMC5990418

[49] Norton T. D. et al. Lentiviral Vector-Based Dendritic Cell Vaccine Suppresses HIV Replication in Humanized Mice. *Molecular therapy*: *the journal of the American Society of Gene Therapy* 27, 960–973, 10.1016/j.ymthe.2019.03.008 (2019). 30962161 PMC6520467

