# Correction: Robust CAR-T memory formation and function via hematopoietic stem cell delivery

**DOI:** 10.1371/journal.ppat.1009895

**Published:** 2021-08-30

**Authors:** Anjie Zhen, Mayra A Carrillo, Wenli Mu, Valerie Rezek, Heather Martin, Philip Hamid, Irvin S. Y. Chen, Otto O. Yang, Jerome A. Zack, Scott G. Kitchen

The following information is missing from the Funding statement: This work was supported by the UCLA AIDS Institute, the James B. Pendleton Charitable Trust, and the McCarthy Family Foundation.
