# Supplementary figures and images for: Robust CAR-T memory formation and function via hematopoietic stem cell delivery
Source: PLoS Pathog. 2021 Apr 1;17(4):e1009404. doi: 10.1371/journal.ppat.1009404 (PMC8016106; doi:10.1371/journal.ppat.1009404)

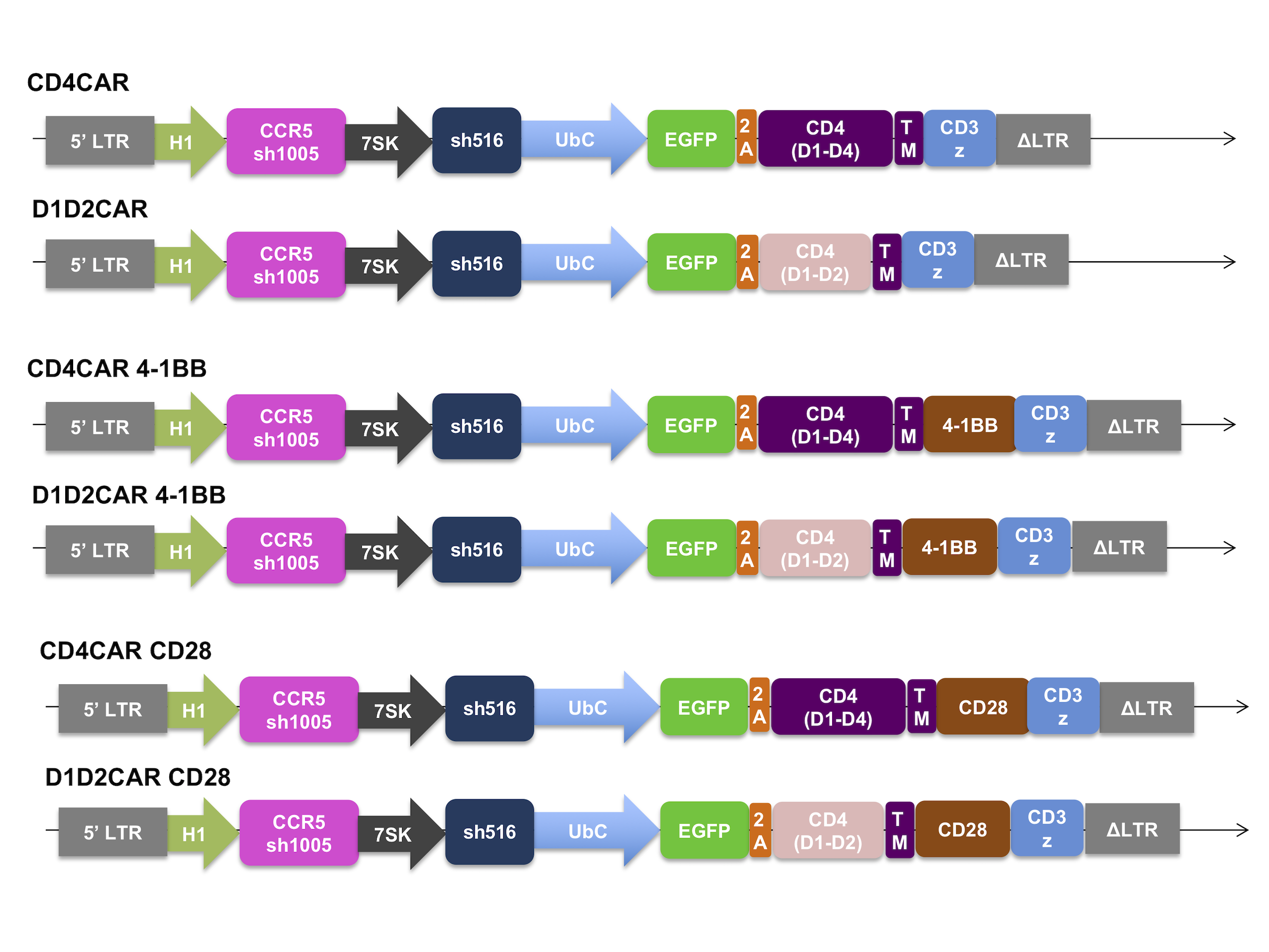

Supplement: S1 Fig — (TIFF) [file ppat.1009404.s001.tiff]

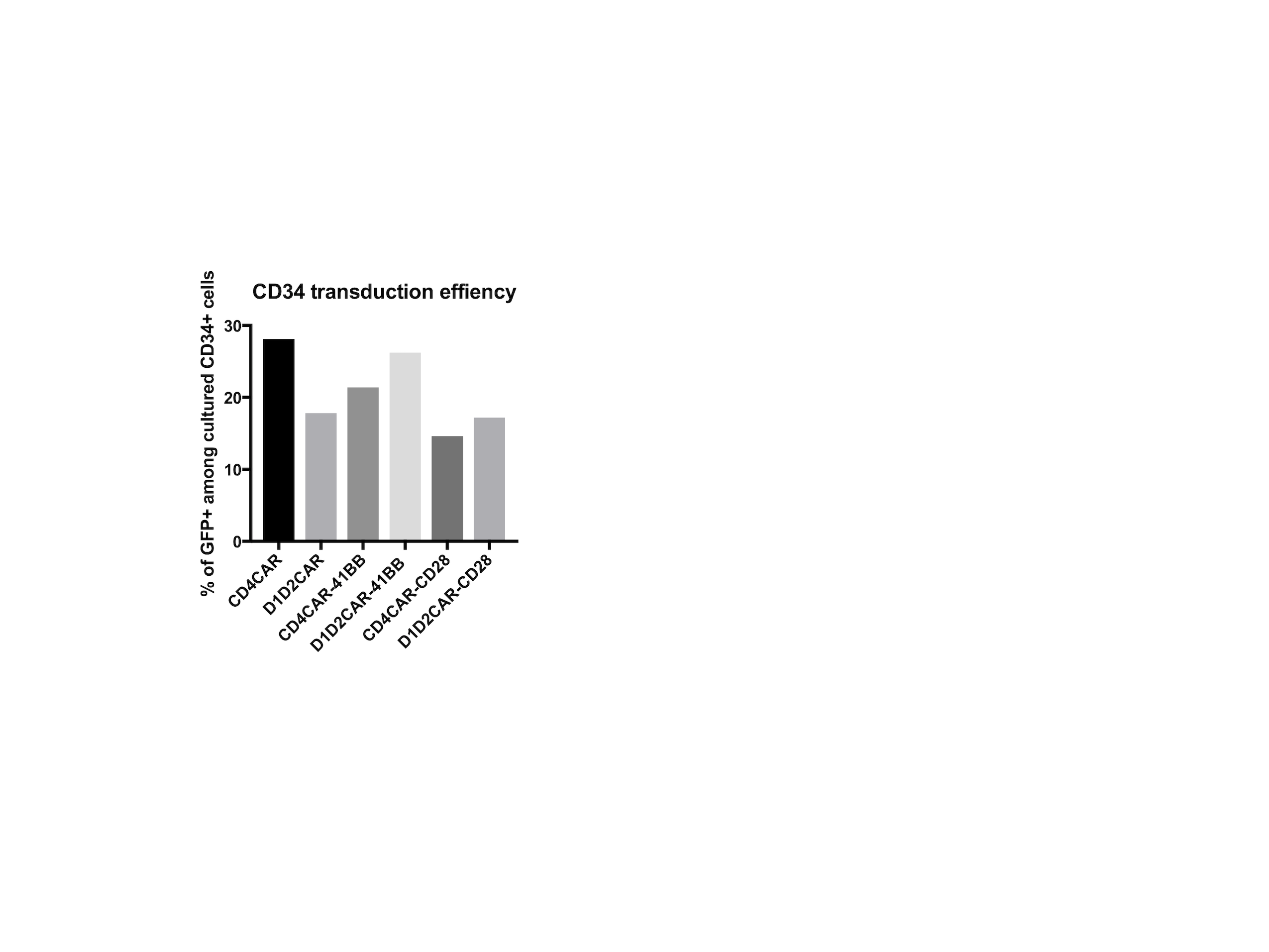

Supplement: S2 Fig — Humanized mice were constructed with donor matched fetal thymus and liver derived CD34+ cells transduced with either CD4CAR, D1D2CAR, CD4CAR 4-1BB, D1D2CAR 4-1BB, CD4CAR CD28 or D1D2CAR CD28. 0.1 million transduced CD34+ cells were set aside from transplant and were cultured in extension culture for 7 days. Afterwards, cells were analyzed by flow cytometry. (TIFF) [file ppat.1009404.s002.tiff]

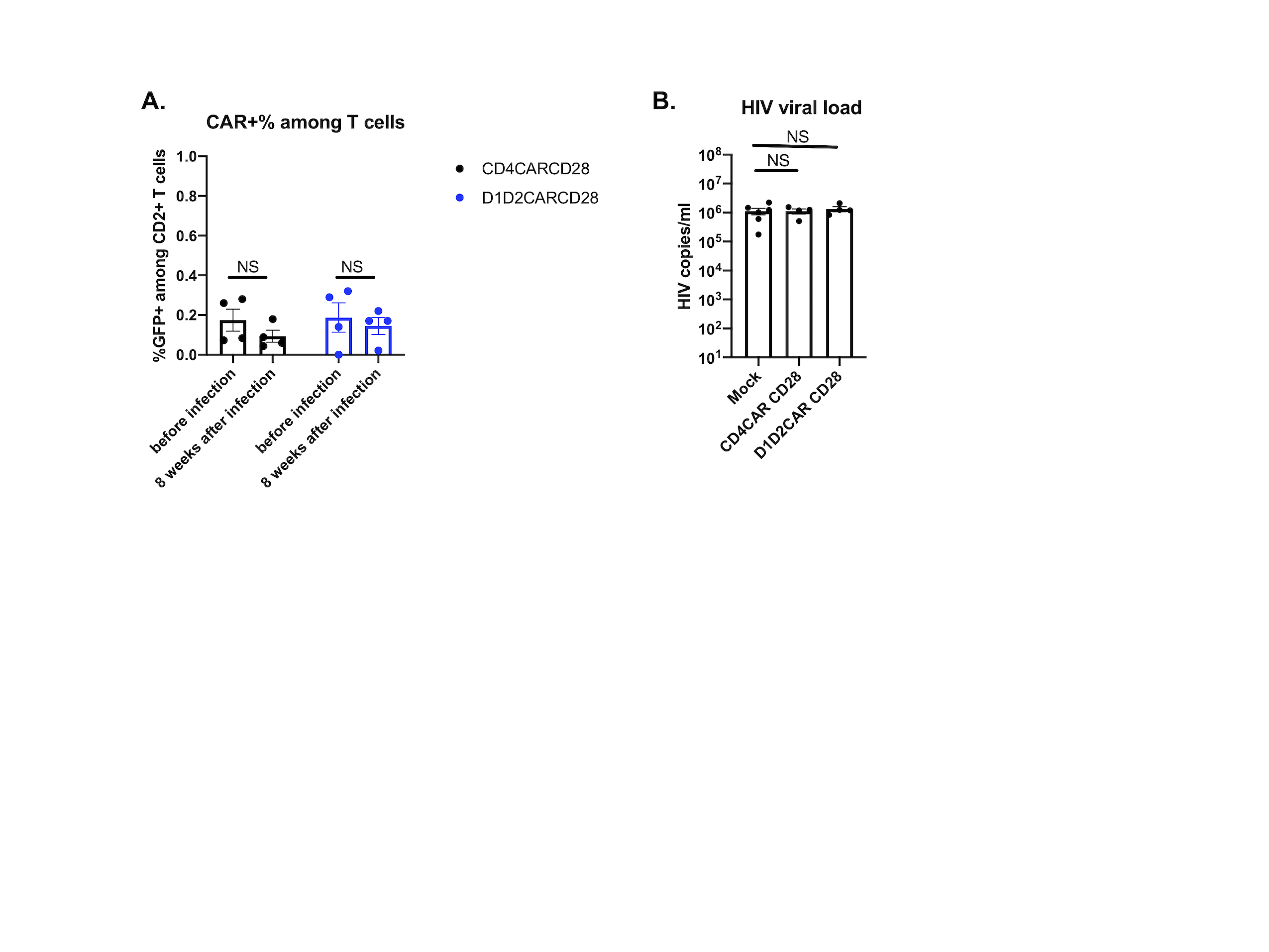

Supplement: S3 Fig — Humanized BLT mice were transplanted with either mock transduced CD34+, or CD34+ cells transduced with lentiviruses CD4CAR CD28 or D1D2CAR CD28. Mice were challenged with HIVNFNSXSL9 after immune constitution. A) GFP+CAR+% among T cells (CD45+CD2+CD56-) were measured before infection and 8 weeks after infection. B) plasma viral load was measured 8 weeks post infection. * p<0.01, **p<0.001, ***p<0.0001 by Mann-Whitney test. (TIFF) [file ppat.1009404.s003.tiff]
